# Crystal structure of a peptidyl‐dipeptidase K‐26‐DCP from *Actinomycete* in complex with its natural inhibitor

**DOI:** 10.1111/febs.13928

**Published:** 2016-11-06

**Authors:** Geoffrey Masuyer, Gyles E. Cozier, Glenna J. Kramer, Brian O. Bachmann, K. Ravi Acharya

**Affiliations:** ^1^ Department of Biology and BiochemistryUniversity of BathUK; ^2^ Department of ChemistryVanderbilt UniversityNashvilleTNUSA; ^3^ Present address: Department of Biochemistry and BiophysicsArrhenius Laboratories for Natural SciencesStockholm UniversityStockholm10691Sweden; ^4^ Present address: Department of BiochemistryUniversity of TorontoTorontoONM5S 1A8Canada

**Keywords:** angiotensin‐I‐converting enzyme, dipeptidyl carboxypeptidase, K‐26 tripeptide, metalloprotease

## Abstract

Several soil‐derived Actinobacteria produce secondary metabolites that are proven specific and potent inhibitors of the human angiotensin‐I‐converting enzyme (ACE), a key target for the modulation of hypertension through its role in the renin–angiotensin–aldosterone system. K‐26‐DCP is a zinc dipeptidyl carboxypeptidase (DCP) produced by *Astrosporangium hypotensionis*, and an ancestral homologue of ACE. Here we report the high‐resolution crystal structures of K‐26‐DCP and of its complex with the natural microbial tripeptide product K‐26. The experimental results provide the structural basis for better understanding the specificity of K‐26 for human ACE over bacterial DCPs.

**Database:**

Structural data are available in the PDB under the accession numbers 5L43 and 5L44.

**Abbreviations**
ACE: angiotensin‐I‐converting enzyme
DCP: dipeptidyl carboxypeptidase

## Introduction

The M3 family of metalloproteases belong to the gluzincin class of enzymes (MEROPS database) 1, which present a single catalytic zinc ion coordinated by the conserved HEXGH‐binding motif. Members of this family are widespread across all organisms, from bacterial dipeptidyl carboxypeptidase (DCP) 2 to the mammalian thimet oligopeptidase 3 and neurolysin 4. These endopeptidases cleave a variety of substrates although generally targeting oligopeptides of less than 20 residues long 5.

*Escherichia coli* DCP (EcDCP) was the first bacterial dipeptidyl peptidase to be isolated 6, 7. It is mostly a cytoplasmic enzyme with a likely role in the degradation of intracellular peptides by removal of the C‐terminal end of its substrates 8. It is strictly confined to a DCP family 7 and is similar to human angiotensin‐I converting enzyme (ACE), which is a major target in the treatment of hypertension and a member of the M2 class of metalloproteases 1. Despite sharing low sequence similarity, many of the binding and active site residues, a large proportion of the secondary structure, and the overall tertiary structural two‐domain arrangement are conserved between these two family members 2. The binding sites of these enzymes have been described using subsites relating to the immediate area around the side chains of the binding peptide ligand. Similarly, the peptide ligand residues are labelled based on the subsite they bind in. The dipeptide released by the enzyme is labelled $P_{1}^{\prime }$ and $P_{2}^{\prime }$, and these residues bind in the $S_{1}^{\prime }$ and $S_{2}^{\prime }$ subsites. The peptide is cleaved between residues P_1_ and $P_{1}^{\prime }$, with residues P_*n*_ binding in subsites S_*n*_.

*Astrosporangium hypotensionis* is a soil bacteria of the actinomycete family that produces a zinc peptidase, K‐26‐DCP, which has strong sequence similarity with its *E. coli* homologue (*E. coli* DCP, 47% sequence identity) 9. *A. hypotensionis* also has an unusual metabolism and is capable of producing complex peptide secondary metabolites. One of such peptides, K‐26, presents a terminal phosphonic acid analogue of tyrosine, (R)‐1‐amino‐2‐(4‐hydroxyphenyl)‐ethylphosphonic acid ((R)‐AHEP) and was first identified through screening for potential bacterial metabolites with inhibitory potency towards human ACE 10. Recent analysis showed the structure–function relationship between K‐26 binding and ACE giving it its specific inhibiting potency on the human enzyme 9. Interestingly, the same study showed that K‐26 is a poor inhibitor of bacterial DCP, including K‐26‐DCP produced by the same organism.

In an effort to further understand the evolutionary aspect of *A. hypotensionis* metabolism and the specificity of the K‐26 peptide and its potential relationship with K‐26‐DCP, we performed a detailed structural analysis of the enzyme by determining its crystal structure in its native form and in the presence of K‐26 at 1.8 Å resolution. Our analysis provides the structural basis of K‐26 recognition by the two homologues DCP and ACE.

## Results and Discussion

### Structure of K‐26‐DCP

**Figure 1:**
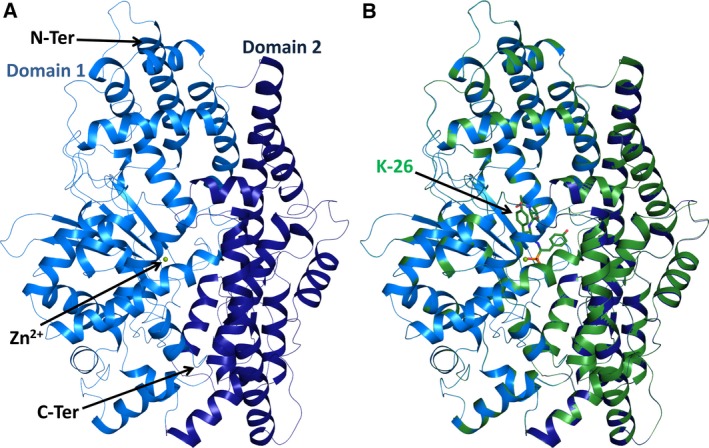
Crystal structure of K‐26‐DCP. (A) Overall structure of apo‐K‐26‐DCP and domain organisation, with subdomain I (light blue) and II (dark blue). The zinc ion of the catalytic site is shown as a green sphere. (B) Overlay of the K‐26‐K‐26‐DCP complex (green) with apo‐K‐26‐DCP (blue). The K‐26 ligand in the complex structure is represented in green stick.

**Figure 2:**
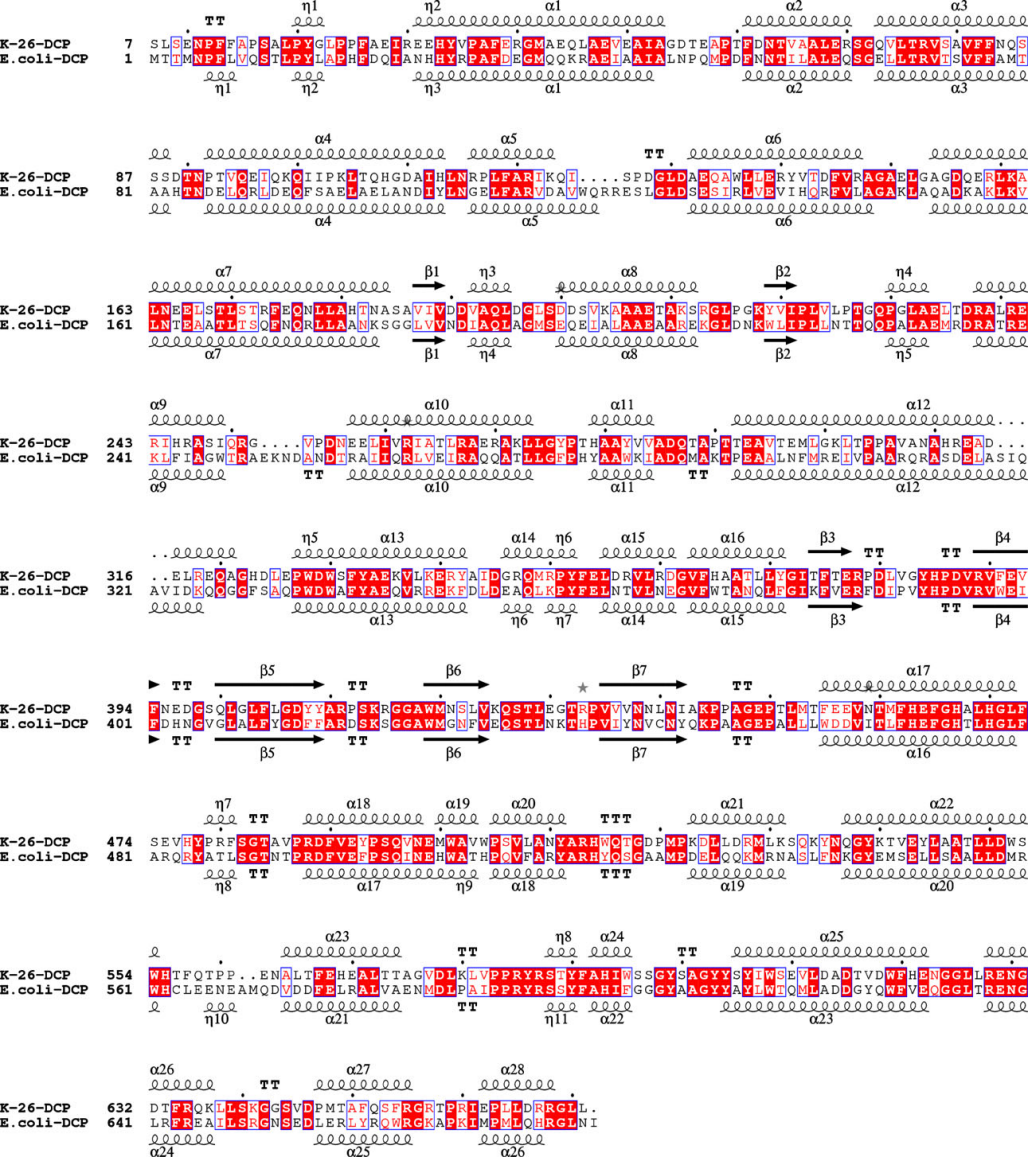
Sequence alignment of K‐26‐DCP with *E. coli* DCP. Sequence and secondary structure alignment produced with ESPript3 23.

**Figure 3:**
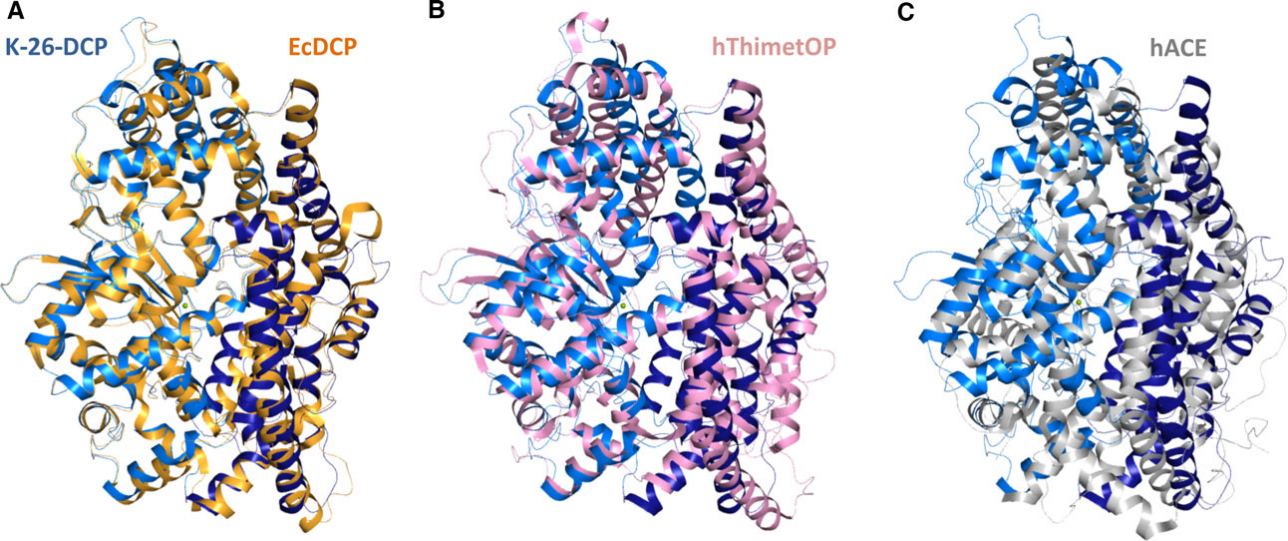
Structural comparison of K‐26‐DCP with other dipeptidyl carboxypeptidases. Superposition of K‐26‐DCP with (A) *E. coli* DCP (orange, PDB 1Y79 2); (B) human thimet oligopeptidase (pink, PDB 1S4B 24) and (C) C‐domain of human ACE (grey, PDB 4BZR 9).

The crystal structures of the DCP from *A. hypotensionis* with and without a bound ligand were determined at 1.8 Å resolution (Table 1). The two crystal structures were solved in the P_1_ space group, each with two molecules per asymmetric unit, which are almost identical (for both structures, the rmsd between the two molecules is 0.12 Å for 662 C^α^ atoms). The apo and the ligand bound structures of K‐26‐DCP superpose with an rmsd of < 0.2Å for 1324 C^α^ atoms. The structure possesses a global prolate ellipsoid shape and was caught in its closed conformation (Fig. 1). K‐26‐DCP (the present structure) superposes well with EcDCP (PDB 1Y79) 2 (rmsd of 0.8 Å over 596 C^α^ atoms) with which it shares 47% amino acid sequence identity (Figs 2 and 3A). The two subdomains are covalently linked in four positions at the back of the molecule and flank the catalytic channel. They are believed to undergo a hinge motion to allow opening of the central cleft, which can then close upon substrate binding 2.

**Table 1:** Crystallographic statistics of the K‐26‐DCP structure and in complex with K‐26

	K26‐DCP	K26‐DCP:K‐26 complex
Resolution (Å)^a^	58.8–1.80 (1.85–1.80)	100.7–1.75 (1.80–1.75)
Space group	P_1_	
Cell dimensions and angles	*a* = 61, *b* = 67, *c* = 102 Å; α = 97, β = 91, γ = 117°	*a* = 62, *b* = 67, *c* = 102 Å; α = 98, β = 91, γ = 117°
Number of molecules/asymmetric unit	2	2
Total/Unique reflections	401 693	244 893
	124 841	128 529
Completeness (%)^a^	95 (62)	89 (55)
*R*_merge_ a ^,^ b	8.4 (62.4)	9.9 (27.7)
*R*_pim_ a ^,^ c	8.4 (62.4)	9.9 (27.7)
<I/σ(I)>^a^	7.3 (1.8)	5.6 (2.3)
CC_1/2_ d	0.99 (0.54)	0.98 (0.81)
Multiplicity	3.2 (2.8)	1.9 (1.8)
*R*_cryst_ e	17.7	18.5
*R*_free_ f	20.0	21.3
Rmsd in bond lengths (Å)	0.011	0.011
Rmsd in bond angles (°)	1.41	1.44
*B*‐factor statistics (Å^2^)		
Protein all atoms (per chain)^g^	21.3/22.6	16.5/15.5
Protein main chain atoms (per chain)^g^	19.7/21.1	15.3/14.3
Protein side chain atoms (per chain)^g^	23.0/24.1	17.6/16.7
Ligand (per chain)^g^	N/A	16.1/16.0
Solvent atoms	30.7	23.9
Zn^2+^/Mg^2+^/SO4^2−^ ions	20.6/20.2/35.8	9.1/ 17.3/ N/A
Ramachandran statistics		
Favoured	98.0%	98.0%
Outliers	0.0%	0.0%
PDB code	5L43	5L44

^a^ Values in parentheses refer to the highest resolution shell. ^b^ *R*_merge_ = ΣΣ_*i*_|*I*_*h*_ − *I*_*hi*_|/ΣΣ_*i*_ *I*_*h*_, where *I*_*h*_ is the mean intensity for reflection *h*. ^c^ *R*_pim_ = Σ_*h*_ (1/*n*_*h*_ − 1) Σ_*l*_|*I*_*hl*_ − (*I*_*h*_ *)*|/Σ_*h*_Σ_*l*_(*I*_*h*_). ^d^ Correlation coefficient between random half data sets [16]. ^e^ *R*_cryst_ = Σ‖*F*_o_| − |*F*_c_‖/Σ|*F*_o_|, where *F*_o_ and *F*_c_ are measured and calculated structure factors respectively. ^f^ *R*_free_ = Σ‖*F*_*o*_| − |*F*_*c*_|/Σ|*F*_*o*_|, calculated from 5% of the reflections selected randomly and omitted during refinement. ^g^ The two *B*‐factor values recorded here correspond to two molecules in the asymmetric unit.

The catalytic site is supported by subdomain I and is composed of the classical HEXXH Zn^2+^ coordination motif, made up by His 463, Glu 464, His 467, and completed by Glu 492. It resides at the centre of the protein deep within the central channel. The structure of the native protein presented unexplained difference density at the active site that could be partly explained by the presence of two sulphate ions in each molecule of the asymmetric unit (see below for further discussion), which was used in the crystallisation medium. No other known components from the experimental environment could be identified. Existence of an unspecific residual peptide as in the case in the *E. coli* DCP crystal structure was ruled out based on careful analysis of the electron density map. Interestingly, two tyrosine residues, Tyr 599 and Tyr 606, reside within close distance of the active site and may play an important role in substrate binding, and stabilisation of catalytic intermediates typical of the general base‐type catalytic mechanism of gluzincins.

K‐26‐DCP structure also possesses conserved secondary structural elements with its closest mammalian homologues neurolysin and thimet oligopeptidase (with 30% sequence identity). Overall K‐26‐DCP is mostly a bundle of 25 α‐helices accompanied by a five‐stranded β‐sheet in subdomain II and a short double‐stranded sheet in subdomain I (Fig. 3B). Additionally, K‐26‐DCP shares some topological likeness with enzymes from the M2 family 1. Despite presenting low sequence identity with human ACE (e.g. 18% with the C‐domain), both families share a common globular fold, size, and subdomain configuration, as seen with the C‐domain of human ACE (PDB 4BZR) (Fig. 3C). Remarkably, subdomain I superimposes particularly well and present similar α‐helical contents. This subdomain holds the conserved catalytic site and the main substrate recognition pockets, and is thus key to the strict DCP activity.

### K‐26 peptide binding to K‐26‐DCP

**Figure 4:**
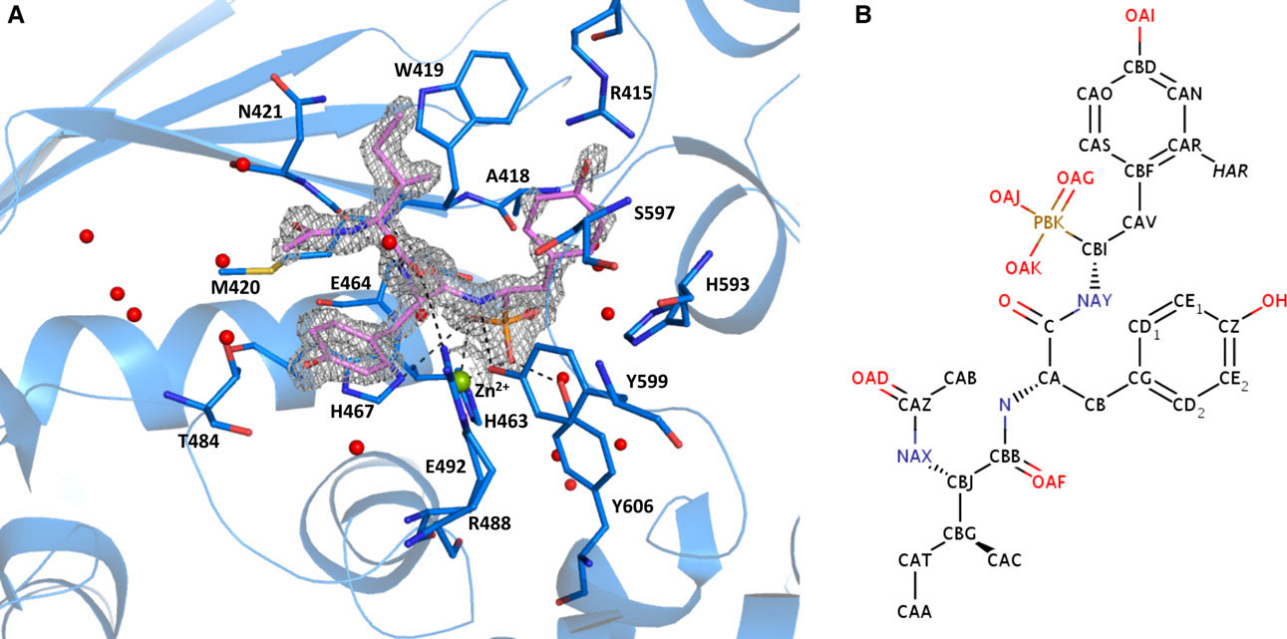
K‐26 ligand binding to K‐26‐DCP. (A). K‐26 (pink) is bound to the enzyme via several electrostatic interactions (black dash) and coordinates the catalytic zinc ion (green sphere). The 2*F*_o_ − *F*_c_ electron density map (2σ) is shown as a grey mesh, water molecules interacting with the ligand are shown as red spheres. (B). Schematic representation of K‐26's chemical structure.

**Figure 5:**
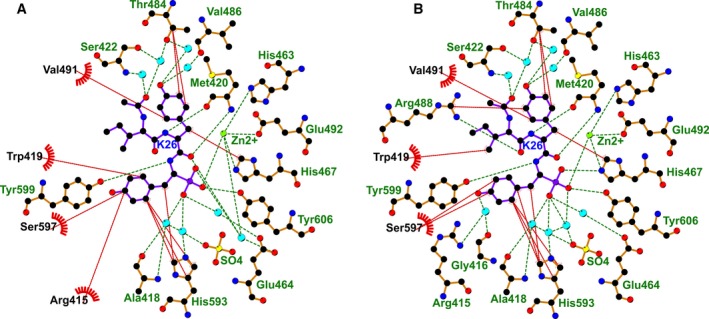
Schematic representation of the K‐26‐binding site in K‐26‐DCP. Hydrogen bond (green dashes) and hydrophobic interactions (red dashes) involved in K‐26 binding to (A) molecule A, and (B) molecule B of the asymmetric unit of K‐26‐DCP crystal structure. Water molecules are shown as blue spheres. The figure was generated using ligplot+ 22.

K‐26 is a phosphonotripeptide produced by *A. hypotensionis* and was first identified based on its high inhibitory potency towards mammalian ACE (IC_50_ = 25 nm) 9. Interestingly, K‐26 was shown to poorly inhibit K‐26‐DCP and its *E. coli* homologue (IC_50_ = 40 and 150 μm respectively) 9. Despite its weak potency, a cocrystallisation experiment showed that the ligand K‐26 (peptide inhibitor) could bind to the active site of K‐26‐DCP. Unambiguous electron density was visible at the S_1_–S_3_ subsites within the substrate‐binding channel (Fig. 4). The protein molecule does not undergo any conformational change upon ligand binding (Fig. 1B). The K‐26 molecule binds in each molecule of the asymmetric unit in the same position and orientation, with only a few differences in the interactions as described below (the following description is for molecule B, with any differences in molecule A highlighted). For both molecules, the potential hydrogen bond interactions of K‐26 binding to K‐26‐DCP are listed in Table 2, all the residues of K‐26‐DCP involved in binding K‐26 are listed in Table 3, and ligplot schematic diagrams of the binding site showing all interactions are shown in Fig. 5.

**Table 2:** Potential hydrogen bonds of K‐26 binding to K‐26‐DCP for each molecule in the asymmetric unit

Residue	A			B		
	Atom	K‐26	Distance (Å)	Atom	K‐26	Distance (Å)
Phosphate						
	Zn^2+^	OAG	2.0	Zn^2+^	OAG	2.1
	Zn^2+^	OAJ	2.4	Zn^2+^	OAJ	2.4
Tyr 606	OH	OAG	2.4	OH	OAG	2.4
His 467	(NE2	OAJ	3.5)^a^	NE2	OAJ	3.3
Water 1	O	OAK	3.3	O	OAK	3.2
Water 2	O	OAK	2.7	O	OAK	2.7
Water 3	O	OAK	2.9	O	OAK	2.8
Water 4	O	OAJ	2.0	–	–	–
Water 5	–	–	–	O	OAG	2.8
	–	–	–	O	OAK	2.9
P_1_						
Water 6	–	–	–	O	OAI	3.3
Tyr 599	OH	NAY	2.9	OH	NAY	2.9
P_2_						
Met 420	N	O	2.8	N	O	2.9
Met 420	O	N	3.1	O	N	3.1
Water 4	O	O	2.3	–	–	–
Water 7	O	OB	3.2	O	OB	2.8
Water 8	O	OB	2.8	O	OB	2.5
P_3_						
Arg 488	(NH2	OAF	3.4)^a^	NH2	OAF	3.0
Water 9	O	OAD	2.9	O	OAD	2.9
Water 10	O	OAD	2.6	O	OAD	2.6

Hydrogen bonds were verified with the program ligplot ^+^ 21. ^a^ Entries in parenthesis are longer than the 3.35 Å limit used by ligplot, but are included for comparison and could form weak interactions.

**Table 3:** Residues involved in K‐26 binding to K‐26‐DCP and ACE homologues

K‐26‐DCP A	K‐26‐DCP B	C‐ACE	N‐ACE A	N‐ACE B	AnCE
R415 (1H)	R415 (1W)	–	–	–	–
–	G416 (1W)	–	–	–	–
–	–	H353 (1W)	H331 (1W)	–	H337 (1D)
A418 (2W)	A418 (2W)	–	–	–	–
W419 (1H)	W419 (1H)	S355 (1H)	S333 (1H)	S333 (1H)	–
M420 (2D)	M420 (2D)	A356 (2D)	A334 (2D)	A334 (2D)	A340 (2D)
–	–	W357 (2H)	W335 (2H)	W335 (2H)	W341 (3H)
S422 (2W)	S422 (2W)	D358 (1D 1W)	D336 (1D)	D336 (1D)	D342 (1D)
–	–	Y360 (1H)	Y338 (1H)	Y338 (1H)	Y344 (1W 2H)
H463 (1Zn)	H463 (1Zn)	H383 (1D 1Zn)	H361 (1Zn)	H361 (1Zn)	H367 (1Zn)
E464 (2W)	E464 (1W)	E384 (1W)	E362 (1W)	–	E368 (1W)
H467 (1D 1Zn 1H)	H467 (1D 1Zn 1H)	H387 (1D 1Zn 3H)	H365 (1D 1Zn 3H)	H365 (1D 5Zn 1H)	H371 (1Zn 4H)
–	–	F391 (2H)	–	–	F375 (2H)
–	–	R402 (1W)	–	–	–
T484 (2W 2H)	T484 (2W 2H)	–	–	–	–
–	–	–	–	–	T387 (1D 2W)
V486 (1W)	V486 (1W)	G404 (1W)	–	G382 (1W)	G388 (1W)
–	R488 (1D 1H)	–	–	–	–
V491 (1H)	V491 (1H)	H410 (4H)	H388 (3H)	H388 (2H)	H394 (3H)
E492 (1Zn)	E492 (1Zn)	E411 (1W 1Zn)	E389 (1W 1Zn)	E389 (1W 1Zn)	E395 (1W 1Zn)
–	–	F512 1 (H)	F490 (2H)	F490 (2H)	Y496 (1H)
H593 (1W 4H)	H593 (1W 5H)	H513 (1W)	H491 (1W)	–	–
S597 (1H)	S597 (2H)	–	–	–	–
–	–	V518 (2H)	–	–	V502 (2H)
Y599 (1D)	Y599 (1D)	–	–	–	–
–	–	R522 (2W)	R500 (2W)	R500 (1W)	R506 (2W)
Y606 (1D)	Y606 (1D)	Y523 (1D 1W)	Y501 (1D 1W)	Y501 (1D 1W)	Y507 (1D 1W)

Residues involved in direct hydrogen bonding (D), water (W) and Zn^2+^ (Zn)‐mediated interactions, and those providing hydrophobic interactions (H) were verified with the program ligplot ^+^ 21. The number of interactions for each type is also indicated.

**Figure 6:**
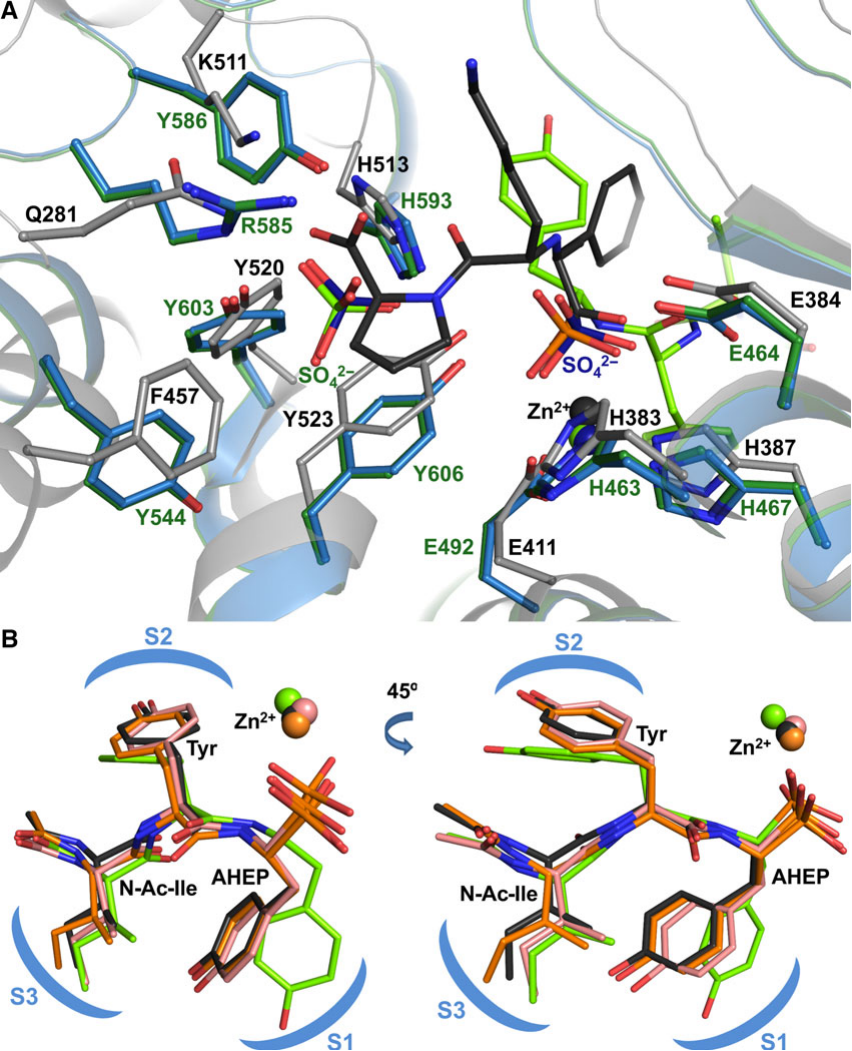
Features of the K‐26‐DCP‐binding site. (A). Overlay of binding sites of apo‐K‐26‐DCP (blue), K‐26‐DCP/K‐26 complex (green) and C‐ACE/lisinopril (grey, PDB 1O86 [25]). Lisinopril is shown as dark grey and K‐26 as bright green. Surrounding side chains are shown as sticks. Zn^2+^ ions are shown as spheres coloured the same as their corresponding protein. Bound sulphates (dark blue for apo‐K‐26‐DCP, bright green for K‐26‐DCP/K‐26 complex) in the K‐26‐DCP structures mimic Zn^2+^ binding and C terminus of peptide ligands. (B). Superposition showing two orientations of K‐26 when bound to K‐26‐DCP (green), C‐ACE (grey, PDB 4BZR 9), N‐ACE (pink, PDB 4BZS 9) and AnCE (orange, PDB 2XHM 11) complex structures. The S_1_–S_3_ subsites are schematically represented. Zn^2+^ ions are shown as spheres coloured the same as their corresponding protein.

The binding pocket clearly showed that the terminal phosphonic acid group directly coordinates the Zn^2+^ ion via two of its oxygen atoms and can make hydrogen bonds with the hydroxyl group of Tyr 606 and the side chain of His 467. Additionally, the third oxygen of the phosphonate moiety is surrounded by a network of four water molecules that interact with residues of the $S_{1}^{\prime }$ subsite. In molecule A, only three of these water molecules are conserved, but there is an additional water molecule that interacts with one of the same oxygen atoms as the Zn^2+^ ion. It is of interest to note that one of the two sulphate ions observed in the active site of the apo‐K‐26‐DCP structure overlays with the phosphonic acid group of K‐26 in the complex structure (Fig. 6A). Similar interactions with the Zn^2+^ ion, and residues Tyr 606 and His 467 are seen, but there are less water molecules in the vicinity. The main chain of the modified peptide is anchored within the binding channel through direct hydrogen bonds with Tyr 599, Met 420 and Arg 488. In molecule A, Arg 488 is located further away from the K‐26 molecule (3.4 Å), such that any potential hydrogen bond would be weaker when compared to the 3.0 Å distance seen in molecule B. But there is an additional water‐mediated interaction with Glu 464.

At the P_1_ position of K‐26, the side chain of the phospho‐tyrosine moiety is stabilised by water‐mediated interactions of its hydroxyl with Arg 415 and Gly 416 (these water‐mediated interactions are not conserved in molecule A), and hydrophobic interactions from His 593 and Ser 597 (Arg 415 and Trp 419 also provide hydrophobic interactions in molecule A). The ligand's central tyrosine is sitting within a small S_2_ hydrophobic patch composed of Thr 484 and Val 491 residues, with an additional hydrophobic interaction from Arg 488 (this is absent in molecule A). The hydroxyl group of this tyrosine extends into the wider part of the channel and makes water‐mediated contacts with Thr 484 and Val 486. The P_3_ position of the ligand and its *N*‐acetyl isoleucine side chain is located within the S_3_ subsite where it appears to make potential weak hydrophobic interactions with residue Trp 419 (this interaction is absent in molecule A). The oxygen atom of the acetyl terminal is stabilised through water‐mediated interactions with the side chains of Ser 422 and Thr 484.

A sulphate ion is also located in the binding site of the K‐26‐DCP‐K‐26 complex structure, which overlays with the second sulphate ion observed in the apo structure. In structures of N‐ and C‐domains of human ACE (N‐ACE and C‐ACE respectively) in complex with peptides, the C‐terminal position of the peptides consistently occupies the same position in the binding site, showing conserved interactions, and many inhibitors also mimic this position. Allowing for structural differences between K‐26‐DCP and human ACE, the sulphate ion observed in K‐26‐DCP is located in a similar position to the C terminus of peptide ligands bound to ACE (Fig. 6A).

### Comparison with the binding of K‐26 to other ACE homologues

K‐26 was shown to bind to both N‐ACE and C‐ACE, and the *Drosophila melanogaster* homologue AnCE (PDBs 4BZS, 4BZR and 2XHM respectively) in a location whereby it solely occupies the ‘non‐prime’ side of the catalytic channel 9, 11, in contrast to other inhibitors and phosphonic tripeptides studied to date 12, 13. It does however present a similar direct coordination of the zinc ion, in this case by the phosphonate group. The entire K‐26 molecule then fills the S_1_–S_3_ subsites of the ACE homologues, remarkably adopting a very similar conformation in both N‐ACE and C‐ACE, as well as in AnCE (Fig. 6B). Interestingly, the pharmacological property of K‐26, in particular the specificity towards human somatic ACE and AnCE (based on a classical substrate cleavage assay) is more pronounced (IC_50_ = 25 and 160 nm respectively) compared with that measured against K‐26‐DCP (IC_50_ = 40 μm) 9, 11. This considerable difference in inhibition between K‐26‐DCP and the other two enzymes led us to compare their structure in complex with the ligand.

**Figure 7:**
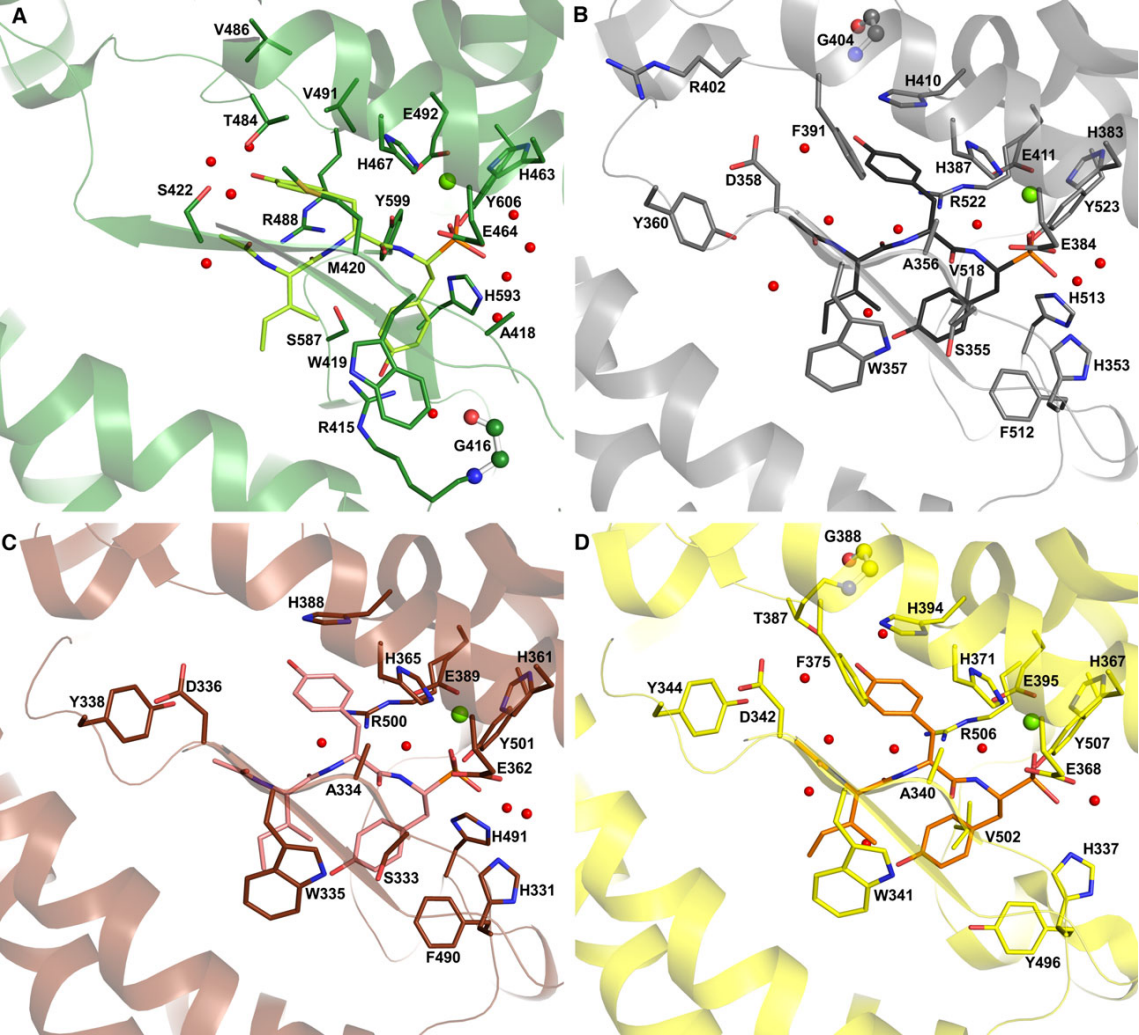
Comparison of K‐26 binding to K‐26‐DCP and ACE homologues. (A). K‐26 (bright green) bound to K‐26‐DCP (green); (B). K‐26 (dark grey) bound to C‐ACE (grey, PDB 4BZR 9); (C). K‐26 (pink) bound to N‐ACE (brown, PDB 4BZS 9); (D). K‐26 (orange) bound to AnCE (yellow, PDB 2XHM 11). Zn^2+^ ions and water molecules are shown as green and red spheres respectively. Residues involved in K‐26 binding are shown as sticks, with only the side chains shown for clarity, with glycine residue backbones shown as ball and stick.

**Figure 8:**
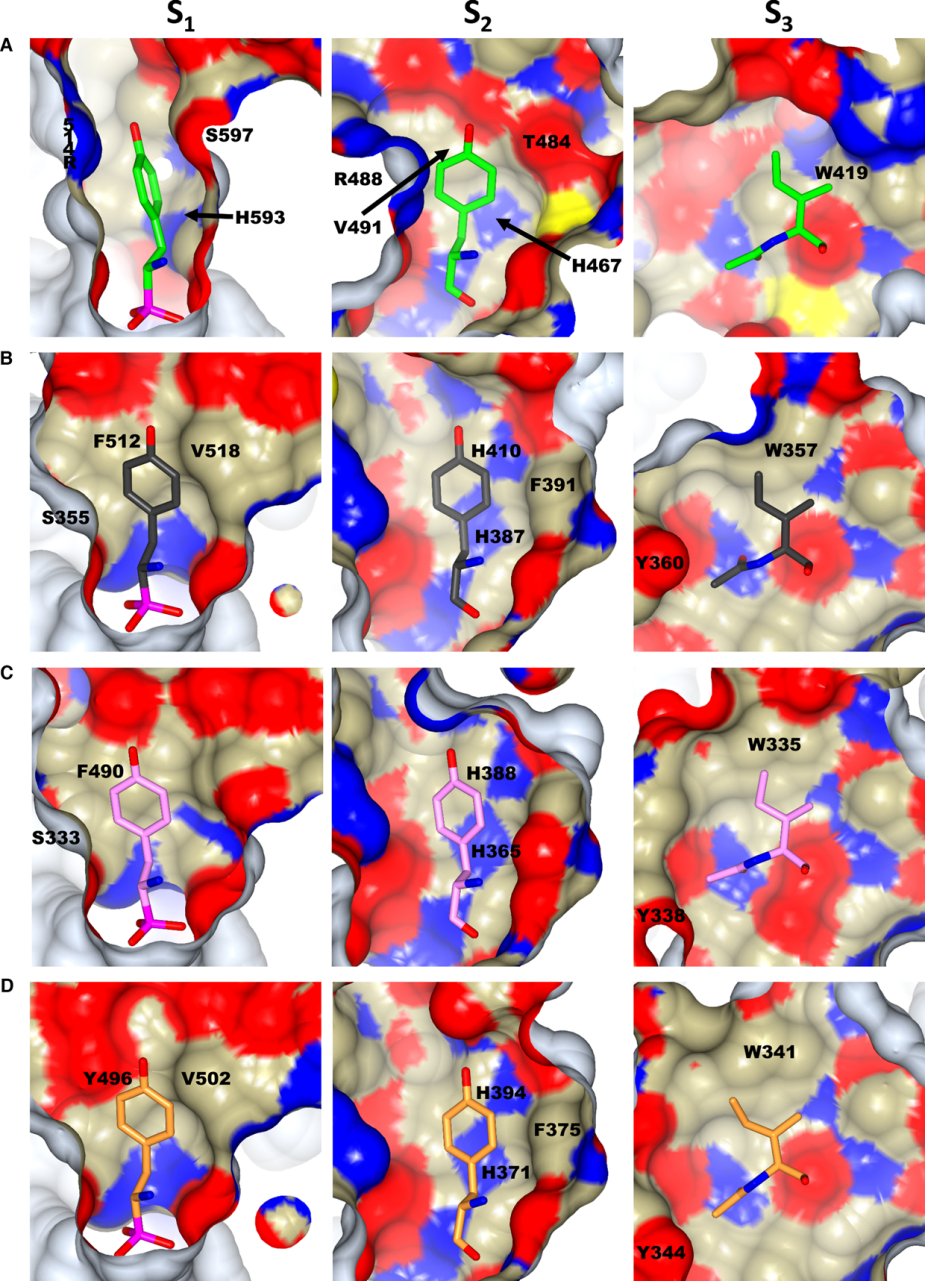
Comparison of K‐26 subsite‐binding environments in K‐26‐DCP and ACE homologues. Internal surface view of subsites S_1_–S_3_ coloured based on atom type, for (A). K‐26‐DCP; (B). C‐ACE (PDB 4BZR 9); (C). N‐ACE (PDB 4BZS 9); (D). AnCE (PDB 2XHM 11). P_1_–P_3_ of K‐26 are shown as sticks, coloured as green for K‐26, dark grey for C‐ACE, pink for N‐ACE and orange for AnCE. Residues involved in hydrophobic interactions are labelled.

At the amino acid level, the two proteins share low sequence identity and show little conservation of residues away from the catalytic site. However, a detailed crystal structure analysis elucidated that K‐26 occupies similar position in K‐26‐DCP to that observed in all the ACE homologue structures, that is being located close to the catalytic zinc ion and extending to the S_3_ subsite (Fig. 7). The side chain of the phospho‐tyrosine occupies the S_1_ subsite, while the central tyrosine fills the S_2_ subsite, and the terminal *N*‐acetyl isoleucine occupies the S_3_ subsite. The main difference between the position of K‐26 in the ACE homologues and in K‐26‐DCP resides in the rotamer conformation of the phosphono‐tyrosine side chain (Fig. 6B). The S_1_ subsite in K‐26‐DCP is much narrower than what is seen in the ACE homologue structures, such that there is not enough space available for the phosphono‐tyrosine to adopt the same orientation (Fig. 8). This causes the tyrosine ring to rotate to an almost perpendicular orientation, and means it is sandwiched between more hydrophilic residues Arg 415 and Ser 597, although there are hydrophobic interactions with Ser 597 and His 593. The hydroxyl group is stabilised by water‐mediated interactions with Arg 415 and Gly 416. However in ACE, its phosphono‐tyrosine side chain has more space available, and is kept in position by hydrophobic interactions (Ser 355, Phe 512 and Val 518 in C‐ACE, Ser 333 and Phe 490 in N‐ACE, and Tyr 496 and Val 502 in AnCE). Although there are hydrophobic interactions with the tyrosine side chain in K‐26‐DCP, the overall environment in the S_1_ subsite is more hydrophobic in the ACE homologues.

This is also observed for the S_2_ and S_3_ subsites in all the ACE homologues, which are noticeably more hydrophobic in comparison to K‐26‐DCP. In addition, there is also less space for the P_2_ tyrosine side chain in the K‐26‐DCP S_2_ subsite when compared to the ACE homologues. This causes a change in its orientation, although not to the same extent as observed for the P_1_ tyrosine. In all the ACE homologues, the P_2_ tyrosine side chain makes a stacking interaction with an aromatic histidine residue (His 410, His 388 and His 394 in C‐ACE, N‐ACE and AnCE respectively). There are additional hydrophobic interactions with the P_2_ tyrosine side chain in all the ACE homologues (His 387 and Phe 391 in C‐ACE, His 365 in N‐ACE, and His 371 and Phe 375 in AnCE). While hydrophobic interactions are observed in K‐26‐DCP, the strong stacking interaction observed in the ACE homologues is replaced with a single interaction with Val 491. The other hydrophobic interactions from His 467, Thr 484 and Arg 488 are less extensive, thus also reducing the overall hydrophobicity of the S_2_ subsite. The S_3_ subsite of the ACE homologues provides a conserved hydrophobic environment, by interactions of the isoleucine side chain with Trp 357 and of the *N*‐acetyl with Tyr 360 in C‐ACE (Trp 335 and Tyr 338 in N‐ACE; Trp 341 and Tyr 344 in AnCE). Phe 391 of C‐ACE (Phe 375 in AnCE) also partly adds to the hydrophobicity around the *N*‐acetyl of K‐26 (Tyr 369 in N‐ACE, therefore less hydrophobic). These residues make it an ideal environment for K‐26's *N*‐acetyl isoleucine. In comparison, the K‐26‐DCP S_3_ subsite only provides a single hydrophobic interaction from Trp 419.

Most of the direct hydrogen bond interactions between protein and K‐26 ligand are conserved in all the structures; in K‐26‐DCP, these are the two backbone hydrogen bonds from Met 420 (the equivalent residue is an alanine in all the ACE homologues, but still providing two backbone hydrogen bonds), directly binding to the zinc ion, and one hydrogen bond each with His 467 and Tyr 606. K‐26‐DCP does have two hydrogen bonds with Arg 488 and Tyr 599 that are not conserved in the ACE homologues, but in turn they have a direct hydrogen bond from an aspartate residue (Asp 358 in C‐ACE), which is not present in K‐26‐DCP. All structures have a selection of water‐mediated interactions. Overall, there is less variation in electrostatic interactions between the different complex structures than observed for hydrophobic interactions.

Thus, the different binding orientation of K‐26 in K‐26‐DCP compared to that seen in the ACE homologues is mainly caused by the more constrained space available in the S_1_ and S_2_ subsites. This, along with the lower hydrophobicity of all subsites in K‐26‐DCP, may explain the difference in inhibitory potency (catalytic efficiency), as the less restricted, more hydrophobic interaction with ACE is more likely to disturb substrate binding than the overall weaker electrostatic interaction with K‐26‐DCP.

## Experimental procedures

### Expression and purification of K‐26‐DCP

The K‐26‐DCP from *A. hypotensionis* was produced by expression in BL21(DE3) *E. coli* cells using the pET28a vector. The N‐terminal His‐tagged protein was purified using a HiTrap nickel affinity column as previously described 9. The concentrated protein was stored at −80 °C.

### Synthesis of K‐26 tripeptide

The K‐26 tripeptide was synthesised as previously described 9.

### Crystallisation and data collection

The crystals of K‐26‐DCP protein with and without K‐26 were grown at 16 °C by the hanging drop vapour diffusion method. K‐26‐DCP was used at 10 mg·mL^−1^ in 50 mm Tris, pH 8.0, 150 mm NaCl. For the complex with K‐26, the protein was preincubated with K‐26 (5 mm) at room temperature for 1 h before crystallisation. Samples were mixed with the reservoir solution consisting of 0.2 m MgCl_2_, 0.1 m sodium cacodylate (pH 6.5) and 20% PEG 8000 and suspended above the well. Crystals of diffraction quality appeared after approximately 2 days. X‐ray diffraction data for crystals with and without the ligand were collected on station IO4 of the Diamond Light Source (Oxon, UK) equipped with a PILATUS‐6M detector (Dectris, Baden‐Dättwil, Switzerland). Crystals were kept at constant temperature (100 K) under the liquid nitrogen jet during data collection. Raw data images were processed and scaled with either dials 14 or mosflm 15, and aimless 16 using the ccp4 suite 6.5 17. Initial phases for structure solution were obtained using the molecular replacement routines of the phaser program 18. The atomic coordinates of the *E. coli* DCP (PDB code 1Y79 2) were used as a search model for structure determination. The resulting models were refined using refmac5 19. Manual adjustments of the model were carried out using coot 20. Water molecules were added at positions, where *F*_o_ *− F*_c_ Fourier difference electron density peaks exceeded 3σ, and potential hydrogen bonds could be made. Validation was conducted with the aid of the program molprobity 21. Crystallographic data statistics are summarised in Table 1. All figures were drawn with pymol (Schrödinger, LLC, New York, NY, USA). Hydrogen bonds were verified with the program ligplot ^+^ 22.

## Conflict of interest

The authors have no conflict of interest to declare.

## Author contributions

GM performed expression, purification of K‐26‐DCP, carried out all the structural biology experiments, analysed the data and wrote the paper. GEC analysed the data and edited the manuscript. GJK performed the initial expression and purification of K‐26‐DCP protein. BOB provided K‐26 tripeptide used in this study, analysed the data and edited the paper. KRA conceived the project, analysed the data and edited the paper. All authors reviewed the results and approved the final version of the manuscript.
